# Modeling the impact of the 13-valent pneumococcal conjugate vaccine serotype catch-up program using United States claims data

**DOI:** 10.1186/1471-2334-12-175

**Published:** 2012-08-03

**Authors:** David R Strutton, Raymond A Farkouh, Jaime L Rubin, Lisa J McGarry, Paul M Loiacono, Keith P Klugman, Steven I Pelton, Kristen E Gilmore, Milton C Weinstein

**Affiliations:** 1 Vaccines Market Access and Outcomes Research Specialty Care Business Unit, Pfizer Inc, 500 Arcola Road, Dock D, COL-D4555, Collegeville, PA, 19426-3930, USA; 2 OptumInsight, 10 Cabot Road, Suite 304, Medford, MA, 02155, USA; 3 Hubert Department of Global Health, Rollins School of Public Health, Emory University, 1518 Clifton Road, N.E – CNR Building Room 6009, Atlanta, GA, 30322, USA; 4 Boston University School of Medicine, 670 Albany Street, Boston, MA, 02118, USA; 5 Department of Health Policy and Management, Harvard School of Public Health, Harvard University, 718 Huntington Avenue, Boston, MA, 02115, USA

**Keywords:** Pneumococcal conjugate vaccine, Catch-up vaccination, Pneumococcal, PCV13, 13-valent

## Abstract

**Background:**

Analysis of US claims data from April 2010 to June 2011 estimated that 39% of the 13-valent pneumococcal conjugate vaccine (PCV13) catch-up eligible cohort would ever receive the catch-up vaccination; a previous analysis assumed 87%.

**Methods:**

This updated figure was applied to a previously published 10-year Markov model while holding all other inputs constant.

**Results:**

Our model estimated that the catch-up program as currently implemented is estimated to prevent an additional 1.7 million cases of disease in children aged ≤59 months over a 10-year period, compared with routine PCV13 vaccination with no catch-up program.

**Conclusions:**

Because 39% catch-up uptake is less than the level of completion of the 4-dose primary PCV13 series, vaccine-preventable cases of pneumococcal disease and related deaths could be decreased further with additional uptake of catch-up vaccination in the catch-up eligible cohort.

## Background

The 13-valent pneumococcal conjugate vaccine (PCV13) has been available in the US for routine vaccination of infants since March 2010. PCV13 provides protection against *Streptococcus pneumoniae* serotypes 1, 3, 4, 5, 6A, 6B, 7F, 9V, 14, 18C, 19A, 19F, and 23F. In February 2010, the Centers for Disease Control and Prevention Advisory Committee on Immunization Practices (ACIP) recommended routine vaccination of all children aged 2–59 months with PCV13, including administration of PCV13 to all children not previously vaccinated with 7-valent pneumococcal conjugate vaccine (PCV7), thereby completing the vaccination series with PCV13 for all children incompletely vaccinated with PCV7, and administration of PCV13 as a fifth dose of conjugate vaccine (a catch-up dose) for all children aged 14–59 months who had been previously fully vaccinated with PCV7 [1,2]. In addition, the ACIP advised that a single dose of PCV13 may be administered for children aged 6–18 years who have not received PCV13 previously but are at increased risk for invasive pneumococcal disease (IPD), including pneumococcal meningitis or bacteremia, regardless of whether they have previously received PCV7 or 23-valent pneumococcal polysaccharide vaccine [1,2].

A recently published model predicted that a PCV13 catch-up program would significantly reduce cases of pneumococcal disease, reduce vaccine-preventable deaths, and increase quality-adjusted life-years within the US population [3]. The analysis indicated that medical cost-offsets would likely be sufficient to cover the cost of the catch-up dose for children aged ≤59 months, making the catch-up program cost-saving under the condition that the catch-up program accelerated establishment of indirect (herd) effects by ≥6 months compared with no catch-up program [3]. A recent report from the Centers for Disease Control and Prevention and a health advisory from California highlight the importance of providing a catch-up dose to eligible children [4,5]. With lower penetration of the catch-up program, it is possible that the acceleration of indirect effect would be <1 year. This short communication presents the estimated impact of the catch-up program with and without indirect effect acceleration, using 15 months of administrative claims data to estimate the catch-up program’s total uptake within the catch-up eligible population.

## Methods

The previous analysis assumed that 87% of eligible children would receive a catch-up dose during the first year of the PCV13 vaccination program [3], equivalent to the percentage of eligible children expected to complete the primary pneumococcal conjugate vaccination series [6]. For the present analysis, the original model was adapted to include the estimate of actual uptake of PCV13 catch-up [3]. To maintain consistency with the previous analysis all other model inputs remained as originally reported [3]. Briefly, the decision-analytic model is a Markov (state-transition) model with the starting point being the choice of vaccination strategy [3]. The 10-year model considers the entire US population, and allows for inclusion of indirect effects. Within each annual cycle, persons are subject to pneumococcal disease, including IPD, all-cause pneumonia (PNE; hospitalized or non-hospitalized), and all-cause acute otitis media (AOM). During each cycle, persons may survive, die from pneumococcal disease, or die from other causes.

Two methods were used to estimate the number of catch-up vaccinations administered to children aged 15–59 months during the observation period of April 2010 to June 2011; age 15–59 months was selected during the model design phase before the ACIP provided its recommendation of 14–59 months. With the first method, catch-up vaccinations in both private and public markets were estimated from the growth in shipments of PCV13 net of customer inventory changes versus historical shipments made in support of the primary PCV7 series. Product wastage was not considered in the model. From this evidence, approximately 24% (3.8 million children) of the age 15–59 month cohort were estimated to have received a catch-up vaccination dose during the 15-month observation period [unpublished data].

A second method was used to verify the private market component results of the first method: private market only catch-up vaccinations were estimated from growth in SDI insurance claims (SDI claims only capture the private market) for children aged 15–59 months during the observation period versus the control period, comprising the average claims in each month over the prior 2 years (i.e. April 2008 through March 2010) [unpublished data]. Insurance claims for office visits within the catch-up eligible cohort were stable over the control period, thus providing a good baseline for comparison. Claims data from SDI (private market only), also predicted an increase in office visits for vaccination of catch-up eligible children during the observation period of 24% [unpublished data].

In order to project the total uptake of catch-up over the eligible period ending in December 2013, a separate model was developed. Using observed increases of vaccination in children aged 14–59 months, age-specific estimates of catch-up participation were obtained via a financial forecasting model. Briefly, the catch-up eligible population at PCV13 launch was based on census birth estimates and National Health Interview Survey-reported 4-dose compliance for PCV7. As time progressed in the model, children were removed from the target population when they became age-ineligible or were vaccinated with a catch-up dose. Age-relevant ‘well’ visit rates and catch-up vaccination rates were extrapolated to estimate the future catch-up uptake of the remaining eligible population. We assumed that as the catch-up eligible cohort ages over time, their probability of receiving a catch-up dose diminishes due to an inverse relationship between age and both vaccination rates and recommended well-visit compliance. The model utilized age-specific SDI claims data applied to the catch-up eligible population [unpublished data]. This model projected final uptake of catch-up among eligible children (aged 15–59 months in March of 2010) to be 39% (6.4 million children).

We estimated cases of IPD, PNE and AOM, and deaths in children aged ≤59 months over a 10-year time horizon assuming different scenarios of uptake levels of the catch-up program. Additionally, we calculated cases of disease and deaths avoided due to catch-up at the present level and those which could be further eliminated with additional uptake of catch-up to 4-dose compliance levels (87%).

The previous analysis assumed that implementation of the catch-up program would accelerate accumulation of indirect effects [3], such that full indirect effects would be realized at 6 years as opposed to the 7 years observed with the introduction of PCV7 vaccination [M.M. Moore: unpublished data]. As we estimated that the lower levels of catch-up vaccination may be inconsistent with the strong acceleration of herd effect assumed in the previous analysis [3], we examined the impact of modifying the assumption of 1-year acceleration through sensitivity analyses. We conducted analyses assuming both 1-year and no acceleration of indirect effects in children aged ≤59 months with the catch-up program, and reported the impact of these assumptions.

## Results

In the base case, the catch-up program as currently implemented is estimated to prevent an additional 1.7 million cases of disease over 10 years in children aged ≤59 months compared with routine PCV13 vaccination with no catch-up program (Table 1). Additional uptake of the catch-up program to 4-dose compliance levels would prevent an additional 0.7 million cases of disease over 10 years compared with current catch-up levels.

**Table 1:** Ten-years disease/mortality, children ≤59 months, multiple PCV13 catch-up scenarios, assuming 6 years to indirect effects

	**PCV7**	**PCV13 with no catch-up**	**Catch-up 39%**	**Catch-up 87%**	**Cases prevented due to catch-up (39%)**	**Additional cases potentially prevented with increased catch-up**^**a**^
IPD	50,762	22,261	20,148	18,822	2,113	1,327
Hospitalized Pneumonia	1,203,733	940,580	916,018	900,426	24,562	15,592
Non-hospitalized Pneumonia	14,828,732	14,037,214	13,958,633	13,905,915	78,581	52,718
AOM	161,033,859	144,731,659	143,125,025	142,466,963	1,606,634	658,062
Deaths	4,389	3,141	3,051	3,020	90	31

*AOM*: acute otitis media; *IPD*: invasive pneumococcal disease; *PCV7*: 7-valent pneumococcal conjugate vaccine; *PCV13*: 13-valent pneumococcal conjugate vaccine.

^a^From 39% to 87%.

In sensitivity analyses, when assuming no indirect-effect acceleration (i.e. 7 years until the accumulation of full indirect effects), the current catch-up program is estimated to prevent an additional 0.5 million cases of disease over 10 years in children aged ≤59 months compared with routine vaccination with PCV13 and no catch-up program (Table 2). Additional uptake of the catch-up program to 4-dose compliance levels is estimated to prevent an additional 0.7 million cases of disease over 10 years compared with current catch-up levels.

**Table 2:** Ten-years disease/mortality, children ≤59 months, multiple PCV13 catch-up scenarios, assuming no acceleration in indirect effects

	**PCV13 with no catch-up**	**Catch-up 39%**	**Catch-up 87%**	**Cases prevented due to catch-up (39%)**	**Additional cases potentially prevented with increased catch-up**^**a**^
IPD	22,261	21,048	19,637	1,176	1,448
Hospitalized Pneumonia	940,580	927,831	912,141	12,749	15,690
Non-hospitalized Pneumonia	14,037,214	13,994,317	13,941,520	42,897	52,797
AOM	144,731,659	144,194,812	143,534,078	536,847	660,735
Deaths	3,141	3,116	3,084	26	32

*AOM*: acute otitis media; *IPD*: invasive pneumococcal disease; *PCV13*: 13-valent pneumococcal conjugate vaccine.

^a^From 39% to 87%.

## Discussion

Our analysis predicts a decline in IPD, PNE, and AOM in children aged ≤59 months with the PCV13 catch-up program. This catch-up program is predicted to have better uptake than the previous PCV7 catch-up program following the introduction of PCV7 [7]. Although a 39% catch-up uptake is less than 87% completion of the 4-dose primary PCV13 series, the program has reached several million children. According to our analysis catch-up has prevented substantial morbidity and mortality due to pneumococcal disease; however, vaccine-preventable cases of pneumococcal disease and related deaths could be decreased further with additional uptake of ACIP catch-up recommendations [2].

Our findings are consistent with the previous analysis which examined disease reduction across all ages [3]. The catch-up program’s estimated cost-effectiveness is unchanged by the percentage of children receiving catch-up; therefore, the cost-effectiveness results, although not discussed in the paper, are also consistent with the previous analysis in that the program remains cost-saving if indirect effects are accelerated by ≥6 months [3].

Under any scenario of catch-up coverage, infants continue to receive high levels of primary series coverage; therefore, we assume that indirect effects caused by PCV13 will be similar to the experience following PCV7. The previous analysis assumed that the indirect effects due to PCV13 would go through a 7-year ramp-up period and that the increased population coverage of the PCV13 catch-up program would accelerate this ramp-up such that the full indirect effects would occur 1 year faster [3]. It is possible that with lower coverage of children aged 15–59 months this acceleration could be muted. This assumption has implications on the reduction of disease in all non-vaccinated cohorts in the early years of PCV13. If catch up is insufficient to accelerate the indirect effect, there is still an increase in the number of cases of pneumococcal disease prevented as cases are reduced in those children who do receive the catch up dose. Most notably, if acceleration of indirect effects occurs with 87% participation, but not with 39% participation, the model estimates an additional 2,263 IPD cases, 115,807 PNE cases, 1.7 million AOM cases, and 95 deaths would occur over 10 years in children aged ≤59 months.

We have understated the benefits of increasing catch-up coverage by excluding individuals aged ≥5 years from the analysis because these individuals benefit from indirect effects. There are additional limitations to this analysis. The model used to estimate the overall uptake of the catch-up program relies on several assumptions. Firstly, the observation period used to project office visits for vaccination is relatively short as PCV13 has been available since March 2010. Secondly, the increase in office visits in the catch-up eligible cohort could be due to other factors that are not yet fully understood in the administrative claims data. Finally, as with any database analysis, we rely on the generalizability of the sample population to the overall US population. Of course, the limitations discussed in the previous analysis also apply to this analysis [3]. However, the claims analysis was remarkably consistent with the secondary PCV13 inventory analysis; therefore, we believe that the 39% estimate is a reasonable estimate of the final uptake of catch-up.

## Conclusion

According to our analysis, catch-up has prevented substantial morbidity and mortality due to pneumococcal disease; however, vaccine-preventable cases of pneumococcal disease and related deaths could be decreased further with additional uptake of ACIP catch-up recommendations [2].

## Competing interests

RF, DS, and PL are employees of Pfizer Inc. JR, LM, and KG are or were employees of OptumInsight, which received financial support from Pfizer in connection with the development of this manuscript. KK and SP have received research funding from Pfizer Inc. MW is a paid consultant to OptumInsight, which has received funding from Pfizer for this study and other research studies.

## Authors’ contributions

DS and RF conceptualized the study. RF designed and conducted all the analyses. PL developed the forecast model to determine catch-up penetration. DS, JR, LM, KG, KK, SP, and MW jointly developed the previously published cost-effectiveness model. JR, LM, KG, KK, SP, and MW analyzed and interpreted the analyses and provided critical feedback on the manuscript. All authors read and approved the final manuscript.

## Authors’ information

Jaime L. Rubin, former employee of OptumInsight.

## Pre-publication history

The pre-publication history for this paper can be accessed here:

http://www.biomedcentral.com/1471-2334/12/175/prepub
